# Development of a comprehensive open access “molecules with androgenic activity resource (MAAR)” to facilitate risk assessment of chemicals

**DOI:** 10.3389/ebm.2024.10279

**Published:** 2024-09-19

**Authors:** Fan Dong, Barry Hardy, Jie Liu, Tomaz Mohoric, Wenjing Guo, Thomas Exner, Weida Tong, Joh Dohler, Daniel Bachler, Huixiao Hong

**Affiliations:** ^1^ National Center for Toxicological Research, US Food and Drug Administration, Jefferson, AR, United States; ^2^ Edelweiss Connect Inc., Durham, NC, United States

**Keywords:** androgen receptor, risk assessment, chemicals, database, open access

## Abstract

The increasing prevalence of endocrine-disrupting chemicals (EDCs) and their potential adverse effects on human health underscore the necessity for robust tools to assess and manage associated risks. The androgen receptor (AR) is a critical component of the endocrine system, playing a pivotal role in mediating the biological effects of androgens, which are male sex hormones. Exposure to androgen-disrupting chemicals during critical periods of development, such as fetal development or puberty, may result in adverse effects on reproductive health, including altered sexual differentiation, impaired fertility, and an increased risk of reproductive disorders. Therefore, androgenic activity data is critical for chemical risk assessment. A large amount of androgenic data has been generated using various experimental protocols. Moreover, the data are reported in different formats and in diverse sources. To facilitate utilization of androgenic activity data in chemical risk assessment, the Molecules with Androgenic Activity Resource (MAAR) was developed. MAAR is the first open-access platform designed to streamline and enhance the risk assessment of chemicals with androgenic activity. MAAR’s development involved the integration of diverse data sources, including data from public databases and mining literature, to establish a reliable and versatile repository. The platform employs a user-friendly interface, enabling efficient navigation and extraction of pertinent information. MAAR is poised to advance chemical risk assessment by offering unprecedented access to information crucial for evaluating the androgenic potential of a wide array of chemicals. The open-access nature of MAAR promotes transparency and collaboration, fostering a collective effort to address the challenges posed by androgenic EDCs.

## Impact statement

The prevalence of endocrine-disrupting chemicals (EDCs) and their potential health impacts necessitate robust tools for risk assessment. The androgen receptor is crucial in mediating the effects of male sex hormones, with disruption during critical developmental periods leading to reproductive health issues. To address this, the Molecules with Androgenic Activity Resource (MAAR) was developed. MAAR integrates diverse data sources to create an open-access platform facilitating chemical risk assessment. By offering easy navigation and extraction of androgenic activity data, MAAR enhances transparency and collaboration in addressing the challenges posed by androgenic EDCs.

## Introduction

Endocrine-active chemicals are exogenous compounds that affect the endocrine system of humans and other vertebrates. Endocrine activity of chemicals has the potential to cause numerous adverse outcomes, including disrupting physiological function of endogenous hormones and altering homeostasis [1, 2]. Evidence that certain man-made chemicals can disrupt the endocrine system by mimicking endogenous hormones sparked intense international scientific discussion and debate starting some 24 years ago [3]. These discussions culminated in issuance of legislation that reauthorized the Safe Drinking Water Act^1^ and authorization of the 1996 Food Quality Protection Act mandating that the US Environmental Protection Agency (EPA) develop a program for screening and testing chemicals with endocrine disrupting potential^2^. In 2015, the US Food and Drug Administration (FDA) published guidance to provide recommendations to sponsors of investigational new drug applications, new drug applications, and biologics license applications regulated by the FDA’s Center for Drug Evaluation and Research (CDER) regarding nonclinical studies intended to identify the potential for a drug to cause endocrine-related toxicity^3^. FDA’s National Center for Toxicological Research (NCTR) developed a program to meet the need for information systems focused on aggregating knowledge of chemicals with experimental results relevant to endocrine activity. These efforts resulted in the development of the endocrine disruptors knowledge base (EDKB) [4] and estrogenic activity database (EADB) [5], which have been used to help identify endocrine active chemicals, develop predictive toxicology models, and prioritize chemicals for laborious and expensive testing [6–22]. However, as of today, androgenic activity data have not been comprehensively curated into a database.

Androgen receptors (ARs) are ligand-dependent transcription factors that belong to the nuclear receptor superfamily [23]. ARs are widely expressed in various tissues within the body [24]. They are the targets for drugs to treat hormone-related diseases including cancers of prostate, breast, ovary, pancreas, etc. [25]. On the other hand, chemicals can interfere with the endocrine system by interacting with ARs, which result in adverse effects [26]. Therefore, estimation of the androgenic activity of drugs and other chemicals is critical for the evaluation of drug safety and assessment of chemical risk.

Over the past decades, large numbers of chemicals have been assayed for androgenic activity by government agencies, industry, and academic research groups, with the results of these studies reported in the public domain. However, the data are distributed across different and diverse sources, obtained in multiple diverse assays, and stored in different formats, limiting the use of the data in research and regulation. Therefore, a comprehensive and reliable resource to provide open access to the data and enable modeling and prediction of androgenic activity for untested chemicals could facilitate advancement in developing strategies to mitigate the AR-driven toxicity and risk. To enable and optimize the use of the data generated by these studies, we have developed and are maintaining a comprehensive open access resource called Molecules with Androgenic Activity Resource (MAAR) to provide the scientific and regulatory communities with an up-to-date androgenic activity database for evaluating potential endocrine activity of chemicals.

## Materials and methods

### Data collection

**FIGURE 1:**
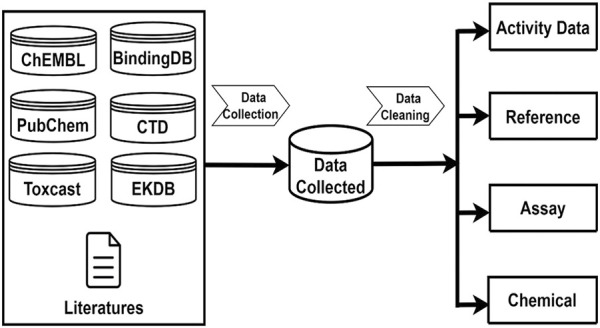
Androgenic Activity Data Collection and Curation. Data collection sources including ChemBL, PubChem, BindingDB, EDKB, CTD, Toxcast. After data pre-processing steps, Androgenic Activity Data are curated into four categories: Activity Data information, Reference information, Assay information, Chemical information.

Androgenic activity data were collected from multiple sources which encompass published literature and public databases including PubChem [27], ChEMBL [28], BindingDB [29], EDKB [4], ToxCast [30], and the Comparative Toxicogenomics Database (CTD) [31]. Java programs were developed to automatically retrieve androgenic activity data points and associated data such as chemical structures, assays, species, and references from these public databases. In addition, androgenic activity data in the literature were manually searched and extracted. The collected androgenic activity data include both quantitative measurements for active compounds and qualitative descriptions for inactive chemicals. Figure 1 gives the sources from which the androgenic activity and related data were collected and the four types of data that were included in this database.

### Data curation

After data were collected from individual sources, data were pre-processed and integrated before they were implemented into the database. Given potential duplications in the data collected from different sources, an automated pre-process program was devised to check and remove duplicated data records by comparing chemicals, activity data, assays, and references. This program identified and removed duplicates by comparing CID, ChEMBL ID, PubMed ID, endpoint values, and assay descriptions across data sources. Geometric and optical isomers are considered duplicates only if they have the same CID or ChEMBL ID. This program also ensured data uniformity by transforming all collected activity data into standardized units. For different activity values of a compound from different sources where inconsistencies were found, a manual review was conducted to determine the most reliable value by examining the assay details. Following a cleaning procedure that removed duplicates to keep unique androgenic activity data, the pre-processed data were combined to make the final data that were included in the database. This program was developed in Java. It processes text file containing all activity information, specifies columns used for comparison, and identifies both duplicate and unique activity records to ensure that non-redundant data is included in the final dataset.

### Data model

**FIGURE 2:**
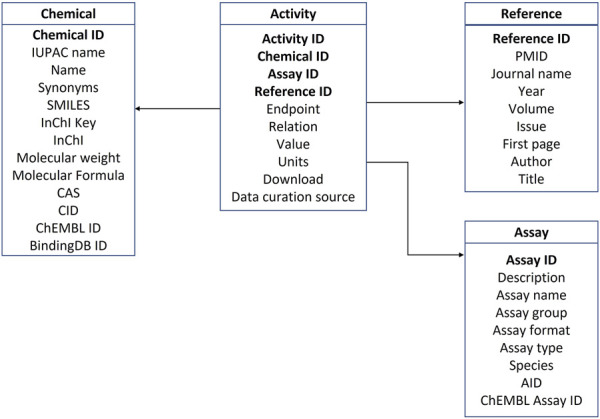
Database schema.

The data implemented in the database were organized into four categories: androgen activity data, references, assays, and chemical information. Properties for each of the four categories are summarized in Tables 1–4. The four tables are interconnected through Chemical ID, Assay ID and Reference ID as depicted in the database schema in Figure 2.

**TABLE 1:** Reference data table.

Data field	Description
Reference ID	Internal ID for reference
PMID	PubMed ID for reference
Journal_Name	Reference journal name
Year	Publication year
Volume	Volume number
Issue	Issue number
First_Page	First page number
Author	Author names
Title	Publication title

**TABLE 2:** Assay data table.

Data field	Description
Assay ID	Internal ID for assay
Description	Description of assay
Assay_Name	Assay name
Assay_Group	Assay group, e.g., HTS, Reporter gene
Assay_Format	Assay format, e.g., cell-based, protein-based
Assay_Type	Assay type, e.g., Agonist, Antagonist
Species	Species assay based on, e.g., *Homo sapiens*
AID	Bioassay AID in PubChem
ChEMBL Assay ID	Assay ID in ChEMBL.

**TABLE 3:** Androgen activity data table.

Data field	Description
Activity ID	Internal ID for activity data
Chemical ID	Internal ID for chemicals
Assay ID	Internal ID for assays
Reference ID	Internal ID for references
Endpoint	Activity endpoint, e.g., IC50, AC50, LogRBA
Relation	Relation to describe activity value, e.g., >, =, <
Value	Activity value from endpoint measurement
Units	Activity data unit, e.g., nM, %
Download	Database where data downloaded, e.g., PubChem, ChEMBL
Curation/Data source	Date source, e.g., literatures, US Patent, Tox21, Abbott Labs

**TABLE 4:** Chemical data table.

Data field	Description
Chemical ID	Internal chemical ID of chemical
IUPAC_NAME	IUPAC name of chemical
Name	Chemical name used in the system
Synonyms	Chemical synonyms (a string separated by “|”)
SMILES	SMILES string of chemical
InChIKey	A fixed-length format directly derived from InChI
InChI	International Chemical Identifier
Molecular_weight	Molecular weight
Molecular_formula	Molecular formula
CAS	Chemical CAS registry number
CID	Compound ID in PubChem
CHEMBL_ID	Compound ID in ChEMBL
BindingDB_ID	Compound ID in BindingDB

### Database design

The curated tables were put into a cloud-based database based on EdelweissData that was developed by Edelweiss Connect, GmbH to tackle data management issues in life sciences. Some of the advantages offered by the EdelweissData solution are:

- Each published dataset is assigned a unique URL and is easily accessible through a web browser.
- Published datasets are automatically versioned.
- Published datasets are static, i.e., they cannot be changed unless a newer version is published.
- Published datasets are immediately available through a web service and can be consumed by numerous data analysis tools (Python, R, Excel, KNIME, etc.) via REST API (see also section Data model).
- Flexibility - there is no predefined schema for published datasets. Instead, the schema is inferred from the data during publishing. This allows for a quick and easy consumption of datasets with various structures.

### Database implementation - EdelweissData

The MAAR Database is built as a simple web application with a back-end supported by EdelweissData and a front-end that lets the user easily explore the database. The most common use cases (such as search by compound or chemical similarity) are well covered by the web application as is, while more advanced queries or analyses could be made through the API (Application Programming Interface) enabled by EdelweissData.

## Results

### Data collected and curated

In total, 125,519 androgenic activity data points for 13,648 chemicals were collected and curated from multiple sources and included in the MAAR database. These data were obtained from 923 assays. Table 5 lists the statistics of the data collected.

**TABLE 5:** Statistics of the data collected in the androgenic activity database.

Chemicals			13,648
Activity data	Quantitative data		48,273
	Qualitative data	Active	723
		Inactive	71,630
		Not determined	4,893
	Total		125,519
Assays	Binding		379
	Reporter gene		358
	Cell proliferation		86
	*In vivo*		60
	HTS		24
	Other		16
	Total		923
Species			6

The androgenic activity data are presented in two types. The first type is quantitative value and 48,273 data points are in this type. A quantitative activity value indicates the androgenic activity is numerically determined. Another type of data is qualitative androgenic activity that is described using qualitative terms: active or positive indicates a chemical was tested using an assay and activity was observed but could not be numerically determined; inactive or negative means a chemical did not show androgenic activity in an assay; inconclusion or not determined or unspecified implies activity of a chemical in an assay was not able to be determined. There are 77,246 data that are qualitative. The data were generated using 923 assays, including 379 binding assays, 358 reporter gene assays, 86 cell proliferation assays, 60 *in vivo* assays, 24 high-throughput screening assays (HTS), and 16 other assays that could not be clearly put into any type of assays. Species used in the activity testing are also included in the database, including *Bos taurus*, Chimpanzee, Chinese hamster, *Homo sapiens*, *Mus musculus*, and *Rattus norvegicus*. Information on species for some assays could not be determined in the sources, and thus they are missed for the data generated using such assays.

**FIGURE 3:**
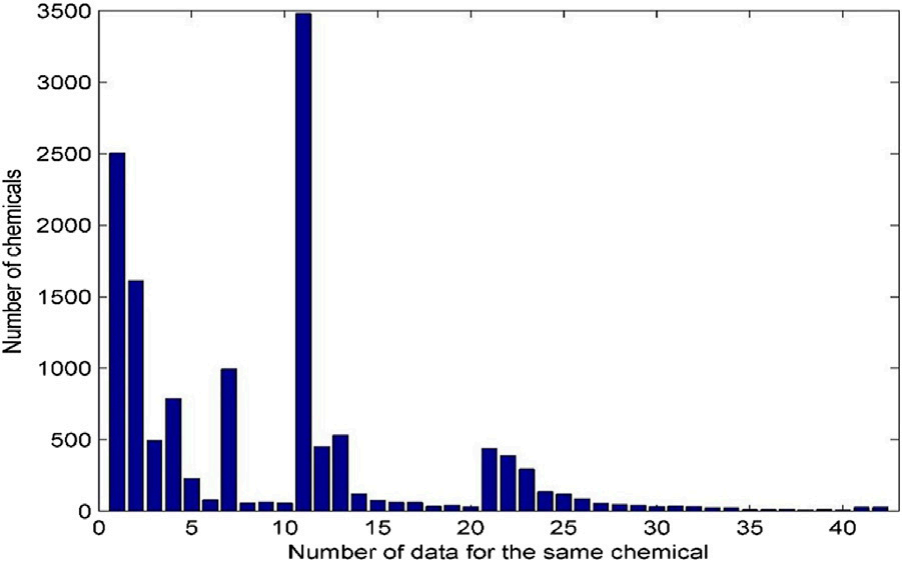
Distribution of androgenic activity data for the same chemical. Each bar represents the number of chemicals. X-axis indicates the number of data records for the same chemicals. The chemicals with 41–50 data records were grouped into the bar with x-axis value 41 and the chemicals with more than 50 data were grouped in the last bar with x-axis value 42.

A chemical could be tested in many laboratories using multiple assays. All androgenic activity data for the same chemicals were collected and presented in this database. The distribution of androgenic activity data for the same chemicals is given in Figure 3.

**FIGURE 4:**
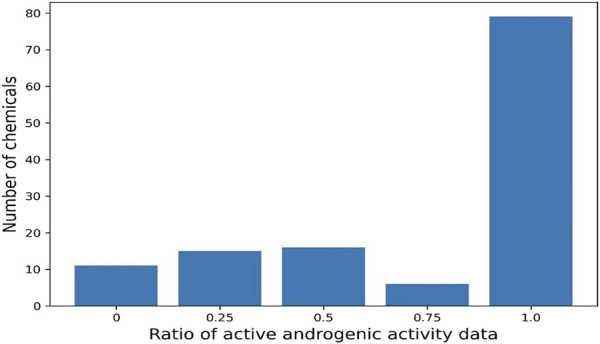
Ratio of active data among 4 binding assay data for 127 chemicals. Each bar represents the number of chemicals. X-axis indicates the ratio of active data.

The same chemical is often tested by a variety of assays and has multiple data records. Of the 13,648 chemicals in the database, 2,504 have only one androgenic activity data and the remaining 11,144 have more than one data. Many chemicals have more than 10 androgenic activity data reported and are included in this database. For example, 3,481 chemicals have 11 data records, and 54 chemicals even have more than 40 data records. The androgenic activity data obtained from the same type of assays in different laboratories could be inconsistent. For example, 127 chemicals each have four androgenic activity data generated using binding assays. As shown in Figure 4, 79 chemicals are active for all four data (100% active) and 11 chemicals consistently show inactive (0% active), while the other (37 chemicals) have inconsistent androgenic activity data: one active and three inactive for 15 chemicals, three active and one inactive for six chemicals, and two active and two inactive for 16 chemicals. This database presents all androgenic activity data reported in different sources. Assessing data quality and selecting data for specific applications such as QSAR (quantitative structure-activity relationship) modelling are critically important. Users should make decisions on how to use the data tailored to their applications.

### Web resource

**FIGURE 5:**
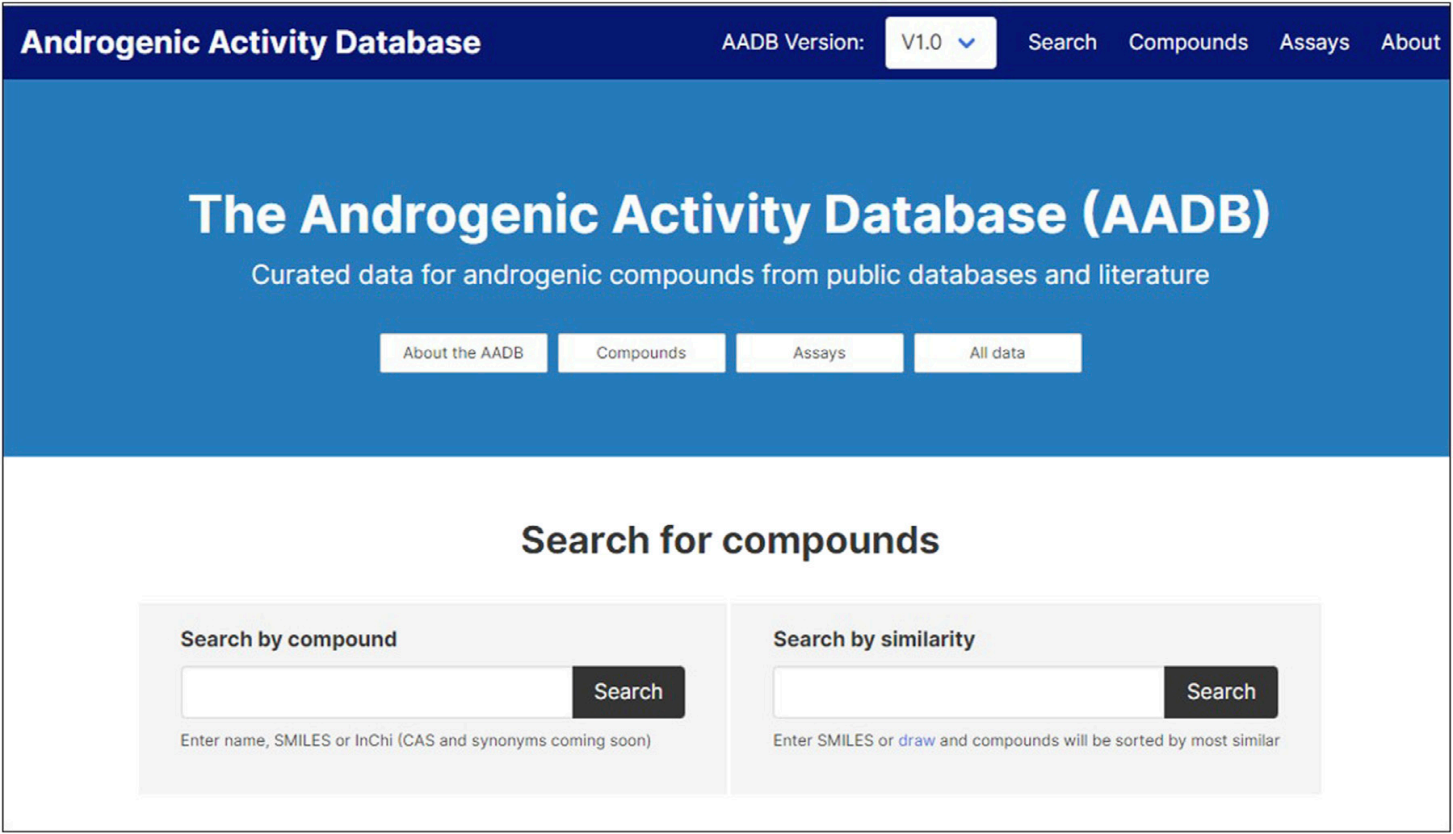
User interface of the web resource.

The MAAR database is made available through a web portal as an open science resource based on open data provided according to a Creative Commons license. We have established the resource as part of the OpenTox open knowledge infrastructure located at^4^. The main initial functionality supported allows the user to search for compounds or chemically similar compounds in the database (Figure 5). The portal also supports the location of community-generated notebooks providing additional analysis of the data, starting with illustrative examples we have provided (see sections Method and Application Programming Interface).

### Application programming interface (API)

The MAAR database comes with a versatile API that simplifies the consumption of the data into other applications. Common data analysis tools that support Representational State Transfer (REST) APIs can obtain data in the database through a simple web request. In this way data can be easily transferred into a Python or R script/notebook, KNIME, Microsoft Excel, etc. To make it even easier for users, an example of an API call in Python and curl^5^ is provided in the web application and could be copy-pasted to the user’s script/notebook. API documentation is available from the EdelweissData main website^6^.

**FIGURE 6:**
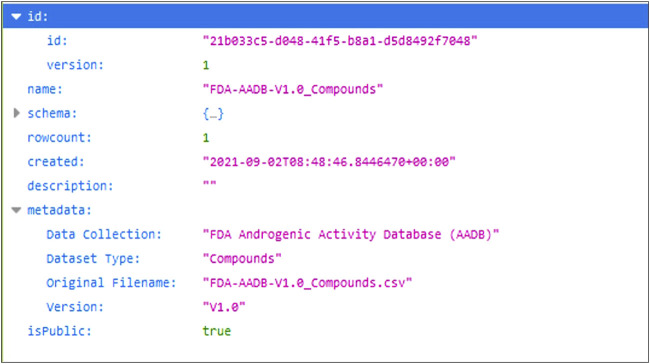
JSON response from the database.

For the purpose of demonstrating programmatic data retrieval from the database, we show an example of how a particular dataset could be accessed with a web request. Each assay dataset in the database has its own unique ID and when the URL pointing to that dataset is called the database returns the dataset in the JSON format. For a dataset inside the database the URL for dataset with ID “21b033c5-d048-41f5-b8a1-d5d8492f7048” would be the following: ^7^. And the response from the database is shown in Figure 6.

### Notebooks

The REST API service mentioned above is very well suited for different interactive notebooks that are nowadays a common tool for data analysis and visualization. There are many different notebooks available today that differ in the language, interactivity, etc. To build an interactive notebook for visualization of the MAAR data we decided to use Observable HQ notebooks^8^ as they offer in our opinion a very good user experience even for technically less skilled users. The programming language in Observable notebooks is JavaScript, which is typically not the language of first choice for data analysis, however, it is very well suited for interactive visualizations that work as a web page.

**FIGURE 7:**
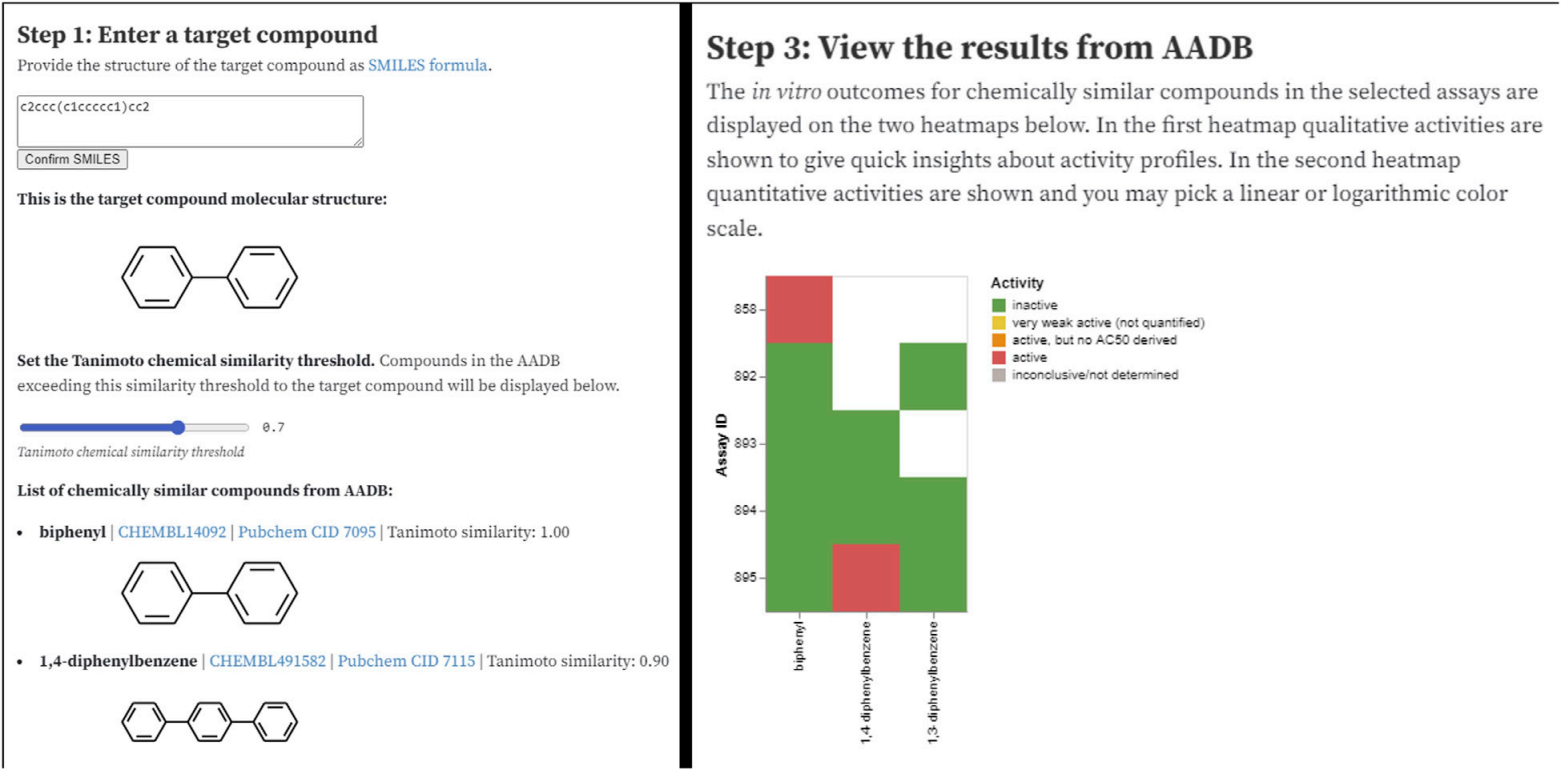
Two sections of the AADB Observable notebook: user interface for entering the input parameters (left) and visualization of filtered results (right).

The Observable notebook^9^ for the MAAR database is available through a URL and can be easily shared with anyone. The notebook addresses a simple use case where a user wants to search the database for a particular compound. The notebook returns a list of chemically most similar compounds (based on the Tanimoto chemical similarity – see Figure 7, left) together with their activities in the assays. In the next step, users can narrow down the set of activities by filtering the assays based on format, group, type, species, or endpoint. Finally, the subset of compounds (on x axis) and their activities (colors) in various assays (y axis) is displayed as a heatmap (Figure 7, right).

## Discussion

Androgens are hormones that play a key role in the development and maintenance of male characteristics. Understanding the androgenic activity of chemicals is important for assessing chemical risk through endocrine disruption. Therefore, androgenic activity data are important for comprehensive chemical risk assessments, providing insight into the potential endocrine-disrupting effects of substances and helping to establish guidelines and regulations to protect human health and the environment.

Vast amounts of androgenic activity data have been generated and reported in the public domain for many chemicals. However, accessing and using androgenic activity data in the public domain may pose several challenges. First, androgenic activity data are contained in different and diverse sources in the public domain. The lack of comprehensive datasets can hinder applications in chemical risk assessment. Second, the importance of data quality and reliability in scientific research cannot be overstated [32, 33]. Sound scientific conclusions rest on the foundation of accurate and trustworthy data. The reliability and accuracy of available androgenic activity data vary. Incomplete or poorly curated datasets can compromise the validity of research findings. Third, androgenic activity data are sourced from various studies, experiments, or databases, leading to heterogeneity in data formats and measurement techniques. Lack of standardized protocols for androgenic activity assessment can make it challenging to compare data from different sources. Finally, without sufficient metadata or contextual information, it may be challenging to interpret and utilize androgenic activity data accurately. Inter-laboratory and species-specific variations in androgenic responses can complicate the interpretation of androgenic activity data. Therefore, to facilitate utilization of the available androgenic data in chemical risk assessment, we aim to develop an open resource of androgenic activity data of molecules so that the huge amount of androgenic activity data generated in the scientific community could be used to accelerate and improve chemical risk assessment.

In this article we report the development of an open science data resource for androgenic activity data. We followed principles established in previous projects including OpenTox^10^, OpenRiskNet^11^ and EU-ToxRisk^12^ [34–41]. The work includes the careful collection and curation of data entering the database and a data model which includes harmonized data to structure the data in a database. Resource functionalities aligned to the FAIR (findability, accessibility, interoperability, and reusability) principles in the preparation and sharing of open science data and supporting further initiatives and use of the project knowledge. We also paid attention to data integrity principles in the construction of the database and the provision of data through harmonized application programming interfaces, supporting the building of web applications making reliable use of the data. This approach should support analysis and modelling goals of the community in making use of the open knowledge resource created by this work.

**FIGURE 8:**
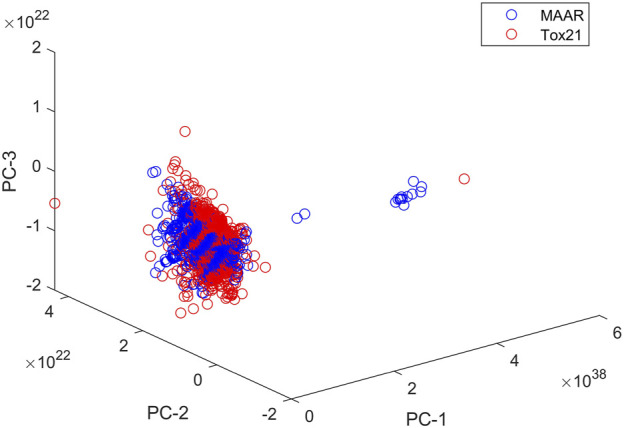
Chemical spaces of MAAR and Tox21. Compounds in MAAR and Tox21 are plotted as blue and red circles, respectively. The x-, y-, and z-axes give the first three principal components.

The MAAR database is an extensive compilation of chemical compounds, systematically curated and annotated for their androgenic properties, providing researchers, regulators, and industry stakeholders with a comprehensive resource for in-depth investigations. To evaluate the structural coverage of chemicals in the MAAR, we computed chemical spaces for both the MAAR and Tox21 [42] datasets using Mold2 descriptors [43, 44]. Following the methodology outlined in our previous studies [19, 45], we performed principal component analysis to represent the chemical space for each dataset. Figure 8 illustrates the first three principal components of the compounds in MAAR and Tox21, demonstrating that the structural coverage of MAAR closely resembles that of Tox21. This comparison confirms that MAAR includes structurally diverse set of compounds, making it suitable for a wide range of applications. Development of the MAAR database represents a significant stride towards a more comprehensive and accessible approach to assessing the androgenic activity of chemicals. By providing a centralized platform for data integration and analysis, the MAAR database is poised to enhance our understanding of androgenic endocrine disruption and contribute to the development of effective risk management strategies in the face of evolving chemical landscapes.

## Conclusions

We have reported here on a useful curated database for androgenic activity provided as an open science resource to the community, and available to enable searches for relevant information on the presence or absence of evidence on androgenic activity of compounds. We have also provided a model and resource with interfaces supporting additional community members to build additional analysis and modelling applications that work with the database. We hope the resource will prove useful and encourage additional development of the resource including addition of new data and its analysis.

## Data Availability

The datasets presented in this study can be found in online repositories. The names of the repository/repositories and accession number(s) can be found below: https://github.com/FANMISUA/AADB.git.

## Footnotes

1
http://www.epa.gov/safewater/sdwa/index.html

2
http://www.epa.gov/scipoly/oscpendo/

3
https://www.fda.gov/media/86996/download

4
https://opentox.net/MAAR/

5
https://en.wikipedia.org/wiki/CURL

6
https://edelweissdata.com/docs/about

7
https://api.aadb.cloud.edelweissconnect.com/datasets/21b033c5-d048-41f5-b8a1-d5d8492f7048/versions/latest

8
https://observablehq.com/

9
https://observablehq.com/@saferworldbydesign/aadb-notebook

10
https://opentox.net/

11
https://openrisknet.org/

12
www.eu-toxrisk.eu
